# Feasibility of an HIV self-testing intervention: a formative qualitative study among individuals, community leaders, and HIV testing experts in northern Tanzania

**DOI:** 10.1186/s12889-020-08651-3

**Published:** 2020-04-15

**Authors:** Bernard Njau, Esther Lisasi, Damian J. Damian, Declare L. Mushi, Andrew Boulle, Catherine Mathews

**Affiliations:** 1 School of Public Health and Family Medicine, University of Cape Town, Cape Town, South Africa; 2 Kilimanjaro Christian Medical University College, Kilimanjaro, Tanzania; 3 Centre of Infectious Disease Epidemiology and Research, University of Cape Town, Cape Town, South Africa; 4 Health Systems Research Unit, South African Medical Research Council, Cape Town, South Africa

**Keywords:** HIV, HIV self-testing, Integrated behavioral model, Northern Tanzania

## Abstract

**Background:**

Achieving the 95–95-95 global targets by 2030, innovative HIV testing models, such as HIV self-testing are needed for people, who are unaware of their HIV status. We aimed to explore key informants, mountain climbing porters, and female bar workers’ attitudes, perceived norms, and personal agency related to HIV self-testing.

**Methods:**

This was a formative qualitative study to inform the design of an HIV self-testing intervention in Northern Tanzania. Informed by the Integrated Behaviour Model, we conducted four focus group discussions, and 18 in-depth interviews with purposively selected participants. Data were analyzed using the framework method.

**Results:**

We recruited 55 participants. Most participants had positive attitudes towards HIVST, in that they anticipated positive consequences related to the introduction and uptake of HIVST. These included privacy and convenience, avoidance of long queues at health facilities, reduced counselor workload, and reduced indirect costs (given that transport to health facilities might not be required). Participants expressed the belief that significant people in their social environment, such as parents and peers, would approve their uptake of HIVST, and that they would accept HIVST. Additionally, features of HIVST that might facilitate its uptake were that it could be performed in private and would obviate visits to health facilities. Most participants were confident in their capacity to use HIVST kits, while a few were less confident about self-testing while alone. Strategies to maximize beliefs about personal agency and facilitate uptake included supplying the self-test kits in a way that was easy to access, and advocacy. Perceived potential constraints to the uptake of HIVST were the cost of buying the self-test kits, poverty, illiteracy, poor eyesight, fear of knowing one’s HIV status, lack of policy/ guidelines for HIVST, and the absence of strategies for linkage to HIV care, treatment, and support.

**Conclusions:**

The findings suggest that HIVST may be feasible to implement in this study setting, with the majority of participants reporting positive attitudes, supportive perceived norms, and self-efficacy. Hence, future HIVST interventions should address the negative beliefs, and perceived barriers towards HIVST to increase HIV testing among the target population in Northern Tanzania.

## Background

In 2015, an estimated 36.7 million people were living with HIV worldwide, and nearly 40% of people living with HIV (PLWH) were unaware of their HIV status [1]. HIV /AIDS is a leading cause of death among Tanzanians, with an estimated 80,000 deaths annually. By 2017, an estimated 1.5 million people were living with HIV, and 83,000 are newly infected each year [1]. In Tanzania, HIV testing services (HTS) are available in more than 2000 sites, including facility-based approaches, which are the most common. Other HTS options, including home-based, mobile or outreach testing campaigns have also been occasionally implemented with significant success. For example in 2007 and 2008: a high-profile nationwide HIV testing campaign attracted more than 3 million people [2]. Additionally, the Tanzanian Ministry of Health and Social Welfare (MoHSW) in 2007 developed guidelines for HTS in clinical settings to compliment the client-initiated testing which had failed to capture important patient groups [3]. Evidence has shown that HTS is an essential component of HIV/AIDS control programs globally and an entry point of the HIV testing cascade. Major benefits associated with HTS include early detection, early initiation of treatment and care, and the introduction of risk reduction strategies for HIV acquisition or onward transmission [1, 4–6].

Despite the benefits of HTS, and the widespread availability of varied HTS options, availability of ART for people living with HIV testing rates remain low in Tanzania. According to the 2016–2017 Tanzania HIV Indicator Survey (THIS), 44.1% of women and 54. 7% of men aged 15–64 years have never tested for HIV. Additionally, only 30% of women and 25% of men tested and received their results in the past year [7].

In the Kilimanjaro region, public health officials have identified two high-risk populations: female bar workers (FBWs) and mountain climbing porters (MCPs). Based on the 2013 Moshi municipal alcohol beverage licensing data, it is estimated that a total of 2000 young women between the ages of 18 and 40 years are working in more than 600 licensed establishments, with additional FBWs working in unlicensed alcohol selling venues. The Tanzanian Business Licensing ACT of 1972 requires the licensing authority to issue a business license only after all FBWs had undergone medical examination and receive medical certificates [8]. Most FBWs are not highly educated, but have a higher level of literacy than average, being single-mothers or divorced. FBWs, who are geographically mobile, choose bar work because it provides an opportunity to earn their income and less dependence on their families [9]. However, in both licensed and unlicensed venues, FBWs have no permanent employment and are paid low wages (e.g., Tanzanian Shillings (TZS) 80,000/= per month (US$ 1 = 2000 TZS)). FBWs remain partially dependent on the financial support of men patrons; some are regular and/ or casual sexual partners. Regular and/ or casual sex partners are distinguished in financial terms. Regular partners provide financial support over an extended period, whilst casual partners pay a predetermined amount of money per single sexual encounter [9]. Existing evidence suggests that apart from serving liquor or beer, FBWs also serve their customers sexually to complement their low income, suggesting that they may be members of core groups at increased risk of HIV infections [9]. There is evidence of 19 to 26% HIV prevalence among FBWs, compared to 5.1% HIV prevalence in the general population [7]. The observed high HIV prevalence among FBWs is associated with HIV risk behaviors, such as excessive alcohol consumption, multiple sexual partnerships, and transactional sexual practices [9, 10]. Evidence suggests that less than 5% of FBWs have never tested for HIV, but 41% of those who have tested have never repeated HIV testing in the past year [11].

An estimated 17,000 porters working in the tourist industry are between the ages of 18 and 45 years but are predominantly young men, mostly coming from poor backgrounds. Also, MCPs are being under-paid, with poor clothing and equipment and shelter, and overloaded making them more vulnerable than their well-equipment clients to fatal health and safety risks [12]. During high seasons (i.e., January to March and June to October), MCPs who are supporting climbers of Mt. Kilimanjaro is very mobile spending extended time away from home and face volatile income cycles. For example, a trip to the top of the Mt. Kilimanjaro takes a minimum of 7 to 10 days depending on the route and the weather. An MCP can climb up to 3 trips non-stop per month to a total of 30 days. Although MCP’s wages are high relative to other unskilled laborers in Tanzania, tips from tourists are a very important supplement boosting their wages by over 50%. The above from the average amount of income may predispose MCPs vulnerable to behavioral risk factors for HIV infection [12]. A descriptive cross-sectional study among MCPs showed behavioral risk factors for HIV infection, including unprotected sex, multiple concurrent sexual partnerships, and substance use, including excessive alcohol consumption, and marijuana use, suggesting a need to motivate sexually active MCPs to engage in HIV prevention interventions [13]. Further, one-third of MCPs have never tested for HIV, despite engaging in high-risk behaviors for HIV infection [11].

Previous studies have documented barriers to accessing HTS in clinical settings, which include stigma and discrimination related to HIV positive results, [14–16] fear of visibility, and lack of confidentiality of HIV positive test result, [17] a lack of privacy, and long waiting time to obtain a test result [18]. HIV self-testing (HIVST) has the potential to circumvent these barriers to reach high-risk individuals in the population [19–21]. HIVST refers specifically to a process in which an individual collects his or her specimen (oral fluid or blood), performs the HIV test and interprets the results, either alone in private, or with assistance from someone they trust [20, 22]. Multiple benefits have been documented that facilitate the high acceptability of HIVST among different target populations [19]. Commonly cited benefits include convenience to test at home, privacy and confidentiality, [23, 24] reduction of stigma due to less visibility, [25] less direct and indirect costs compared with going to an HIV testing point [26]. Also, the features of HIVST that have been regarded as benefits are that it is easy to use, results are obtained in a short time, it is painless, and the non-invasive nature of oral-fluid (OF) tests [27].

HIVST has shown the potential for reaching high-risk populations, including young people and enabling them to know their serostatus [21, 23, 24]. In 2012, the US Food and Drug Administration [28] approved an oral HIV self-test: OralQuick available over-the-counter for HIV self-testing [28]. The approval of the HIV self-test generated self-testing initiatives, particularly in high-income countries, with limited evidence from low- and middle-income countries (LMICs). To support the self-testing initiatives, the WHO developed guidelines for HIVST and partner notification, indicating that HIVST should be recommended as an additional testing option to the existing HTS [20].

The purpose of this qualitative research was to inform the design of an HIVST intervention for FBWs and MCPs in Northern Tanzania. In preparation for the development of the HIVST intervention, we conducted in-depth interviews (IDIs) among key informants, and focus group discussions (FGDs) among FBWs and MCPs to explore their attitudes, perceived norms, and factors affecting their beliefs about personal agency towards the uptake of HIVST.

## Methods

### Theoretical paradigm

We used the Integrated Behavior Model (IBM), to identify the key specific beliefs that best explains their attitudes, perceived norms, and personal agency towards HIVST. IBM is composed of three key constructs which are: (i) attitude towards the behavior, (ii) perceived norms and (iii) personal agency [29]. IBM further categorizes attitude into two components: (i) experiential, which refers to a person’s emotional reaction to the idea of performing the behavior, and (ii) instrumental, which is defined as individuals’ belief about the anticipated positive or negative consequences related to the recommended behavior [30]. The construct “perceived norm” is categorized into two components: (a) injunctive norm, and (b) descriptive norm. An injunctive norm refers to an individual’s belief about the extent to which significant others expect them to do or not to do the recommended behavior. A descriptive norm refers to an individual’s perception of what peers or significant others (i.e., parents, spouses, relatives, religious leaders, etc.), do regarding the recommended behavior [31]. Finally, the construct personal agency is also categorized into two components: (1) self-efficacy, and (2) perceived control. Self-efficacy is the confidence an individual has about his or her capacity to perform the desired behavior, while perceived control refers to an individual’s perceived likelihood of occurrence of each facilitating or constraining condition and beliefs about the effect of facilitators and barriers to behavioral performance [29, 31, 32]. The IBM postulates that a person’s intention to perform a behavior and ultimately the performance of the behavior (e.g. HIV self-testing) is influenced by their attitudes, perceived norms, and personal agency [29].

**Fig. 1:**
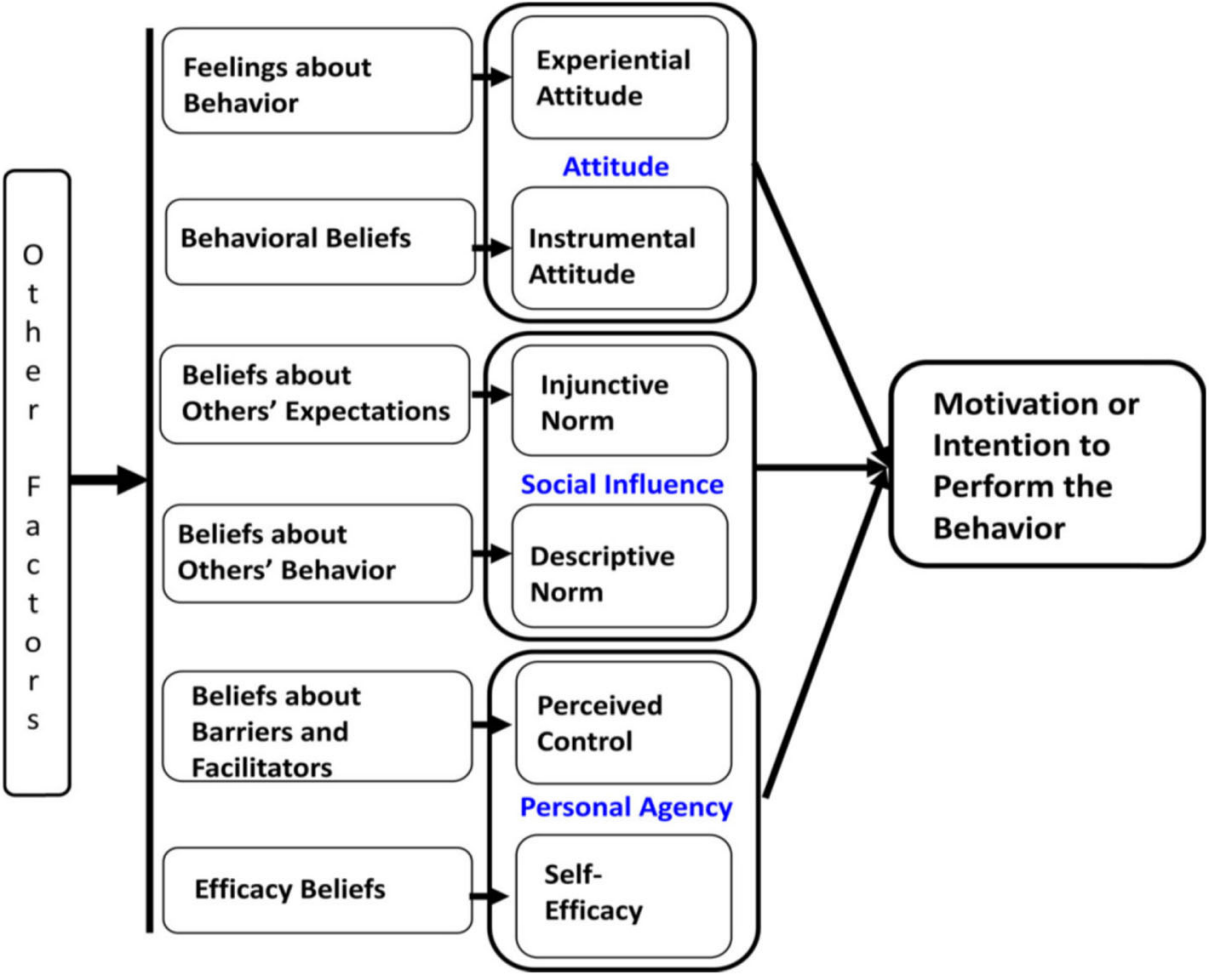
Integrated behavior model

The IBM was adopted in this study because it includes constructs from other influential behavioral theories such as the Theory of Reasoned Action (TRA), the Theory of Planned Behavior (TPB), Social Cognitive Theory (SCT), and the Health Belief Model (HBM), all of which emphasize that value and expectancy beliefs guide behavior [30, 31, 33–35]. Existing evidence suggests that the IBM framework focuses on determinants associated with HIV prevention behaviours, such as HIV testing [35–38] (Fig. 1).

We set out to explore among MCPs and FBWs in Northern Tanzania how the IBM constructs were related to HIVST. This would inform the design of an HIVST intervention for these populations. For this purpose, we explored key informants, FBWs’ and MCPs’ attitudes, perceived norms, and beliefs about personal agency related to the uptake of HIV self-testing.

### Study design and setting

This qualitative formative study was conducted between February 2017 and August 2017 in an urban setting in Northern Tanzania with an estimated population of 202,379 inhabitants [39]. Urban Moshi covers 59 km^2^ (23 sq. m) and is one of the seven administrative districts of the Kilimanjaro region. The tribal groupings residing in the study area are the Chagaa and Pare. The main language used in the study setting is the national language, Kiswahili, spoken by most Tanzanians. The study area is on the tourist circuit, including bars, tourist hotels, major national parks, and the highest mountain in Africa, Mt. Kilimanjaro. At the time of the study, there were 51 health facilities in Moshi urban, which included hospitals (*n* = 3), health centers (*n* = 6), dispensaries (*n* = 14), and freestanding VCT facilities (*n* = 2). Of these health facilities, 17 were public, 3 private for-profits, 3 faith-based organizations, and 2 Non-governmental organizations. Besides, eight HIV Care and Treatment Centers (CTCs) provided access to confirmatory tests and antiretroviral therapy for clients who tested HIV positive [40]. Other HTS included HIV testing campaigns and mobile outreach testing available at venues such as schools, markets, workplaces, etc. In 2007 and 2008 a nationwide HIV testing campaign attracted more than 24,000 testers in the study area [2].

### Study population

The study populations were HIV experts, HTS counselors, community leaders, clinicians, and MCPs and FBWs.

### Sampling and consent

We conducted IDIs with KIs- HIV experts, HTS counselors, community leaders, and clinicians. Subsequently, we conducted FGDs with MCPs and FBWs, some of whom had previously tested for HIV and others who had never tested. Purposive sampling, [41] was employed to recruit 18 key informants as IDI participants (male = 12; females = 6), with varying socio-demographic (e.g., sex, age, occupation, education, marital status, HIV testing experiences, etc.). The sampling approach was chosen to ensure the inclusion of a variety of viewpoints and diverse experiences among participants. HIV experts were recruited at their respective workplaces; clinicians were recruited at local public, and private health facilities; influential leaders were recruited through local government structures. Subsequently, MCPs and FBWs were sampled and recruited and we conducted four FGDs. In the sampling, we stratified by gender and HIV testing status (previously tested for HIV vs. never tested for HIV) and aimed at having between 6 and 12 participants per FGD. In total 37 (MCPs = 21; FBWs = 16), participated in FGDs, with 6 to 12 participants per group. FGD participants were recruited through door-to-door contact in randomly selected bars and tourist companies in Urban Moshi. All participants were 18 years old and above and able to provide consent. Participants were informed that participation in the study was voluntary and that they were free to withdraw at any point. Participants were assigned numbers to ensure anonymity and confidentiality and no personal identifiers were included in the collected data.

### Focus group discussions and in-depth interviews

#### Tool for data collection

Interview guides prepared in English were translated to Kiswahili by a sociologist competent in both languages. Each FGD and IDIs were conducted in Kiswahili-the the national language spoken by most Tanzanians. Based on the IBM constructs, semi-structured guides with open-ended questions were developed for this study for IDIs and FGDs. This approach allowed interviewers to tailor questions and probes as needed for the different participants. The open-ended questions also allowed participants to elaborate on issues considered important or relevant to them. Sample interview guide questions related to the IBM constructs included (Additional file 1).

1. Experiential attitudes: *“How do you feel about the idea of HIVST?*”
2. Instrumental attitudes: *“What are the negative/ or positive consequences that might result from you doing HVST?”*
3. Injunctive norms: “*What would people who are important to you (e.g. parents, spouses, relatives, peers, religious leaders, etc.) think you should do regarding HIVST?”*
4. Descriptive norms: *“What would people who are important to you (e.g. parents, spouses, relatives, peers, religious leaders, etc.) think about HIVST?”*
5. Perceived control: *“What do you think will make it easy or hard for you to do HIVST?”*
6. Self-efficacy: *“How confident are you that you would self-test for HIV?”*

Before the actual data collection, the interview guides were pretested to check for clarity and internal consistency. Eight participants (4 males; 4 females) meeting the same eligibility criteria as the main study participants were involved, which informed minor word use and phrasing adjustments to the final tools.

##### Procedure for data collection

IDIs were conducted at the respective enrollment venues of KIs (i.e., workplaces, public/private health facilities, and local administrative structures, etc.), which lasted approximately 45 min, and were tape-recorded. The FGDs were conducted at a research center in the vicinity of participants’ workplaces, lasted approximately 60 min, and were audio-recorded. A brief orientation of oral-fluid based HIVST was presented during FGDs but participants were not offered the test kits. During the FGDs, notes were taken by an experienced recorder and expanded immediately after the FGDs. The first author [BN] and two-trained research assistants (1 male; 1 female) conducted the FGDs and IDIs using interview guides in Kiswahili. The interview guides had some prompts, which were seldom used because the facilitator wanted to make the interviews as open as possible to gain from the detailed description. Instead words such as “tell me why”, “tell me how”, “tell me more” etc. were frequently used.

Participants were allowed to respond spontaneously to each question and were not required to seek permission to speak or speak in a designated order. Although each participant was not required to answer each question, the facilitator did encourage participation from all participants and made efforts to elicit diverging perspectives. At the end of each interview or discussion, the facilitator presented a quick summary to be confirmed or rectified by the interviewee(s).

The research team met at the end of each discussion for reflection and a summary that described participants’ attitudes, perceived norms, and beliefs related to the uptake of HIV self-testing, and HIV testing experiences was completed. Differences between members of the research team in making meaning of what was observed or heard were resolved after extensive discussions. The summaries help to contextualize the data during data synthesis described below.

#### Data management and synthesis

We used the Framework Method for data management and synthesis. The Framework Method is a systematic and flexible approach and appropriate for analyzing qualitative data. Although the Framework uses a thematic approach, the method also allows themes to originate both from research questions and from narratives of participants. The Framework Method identifies commonalities and differences in qualitative data, focusing on relationships between different parts of the data. Data is analyzed to ensure that commonalities or differences between individuals and groups as units are visible. The Framework Method is divided into seven (7) phases, which provides the procedure for qualitative data analysis. The seven steps include (1) transcription, (2) familiarization of information, (3) coding, (4) developing the analytical framework, (5) applying the analytical framework, (6) charting data into framework matrix, and (7) interpretation of data. The purpose of using the framework approach is to draw descriptive and/or explanatory conclusions clustered around themes, to address the research question and the study aim by producing credible and relevant findings [42]. In this study, the application of the framework method was as follows. In the first step, an independent translator translated the transcripts from Swahili into English. In the second step, two investigators independently examined the transcripts to ensure comparable formatting, until they were satisfied that any inconsistencies in formatting had been resolved. All transcripts were checked for errors by listening back to the audio-recordings and reading the transcripts simultaneously. In the third step, two investigators thoroughly read and re-read each transcript to become familiar with the whole data set. In the fourth step, two investigators reviewed three translated transcripts and applied codes based on the interview questions and probes, and an independent qualitative analyst verified the coded data. Discrepancies in coding were discussed among the three investigators and resolved through consensus. All three investigators agreed on a set of codes, each with a brief definition, which formed the initial analytical framework. In the fifth step, two investigators independently coded three more transcripts using the initial framework, taking care to note any new codes or impressions, which did not fit the existing set. The texts and the codes were read repeatedly to identify pre-defined themes based on the IBM theoretical model and emergent themes from the data. Thereafter, the three authors met to revise the initial framework to incorporate new and refined codes. The process of data refining, applying, and refining the analytical framework was repeated until no new codes were generated, and finally, a formal codebook was created, including a description and example of each code. These codes were based on recognizing forms of responses across data and addressing the aspects of the broader IBM theoretical model. The final framework consisted of pre-defined themes, and codes (Table 1). This process allowed us to generate an interpretive code list, which we used to code all of the remaining transcripts. We attempted to maximize validity by translating the English version interview guides in Swahili, back-translating into English and piloting testing in Swahili before data collection. Furthermore, notes were taken and records of the data collection process and consistent coding of the data to minimize the chance of getting invalid data. This process was used to ensure analytical rigor, and triangulation was ensured through joint analysis of the field notes, collected data and in-person discussions of researchers [43].

**Table 1:** Summary of pre-defined themes and codes from IDIs and FGDs

Pre-defined themes	Codes
**Attitude about HIVST**	
Experiential attitude	Emotional response related to the uptake of HIVST including enjoying the freedom to test oneself, relief to avoid needle pricks, and fear of seeing blood.
Instrumental attitude	Attitude towards HIVST, with anticipated positive consequences, including self-testing at a place of one’s choice, privacy, and convenience, avoiding long queues; reducing time visiting health facilities; less testing and waiting time for test results; reducing counselor’s workload; reducing indirect costs.
**Perceived norms towards HIVST**	
Injunctive norms	A belief that significant people in their social environment, such as parents and peers, would approve (or disapprove) the use of HIVST.
Descriptive norms	An individual’s belief about whether significant people in their social environment, such as parents and peers would use (or not use) HIVST.
**Personal agency towards HIVST**	
Perceived control	**Control belief:** A perceived likelihood that HIVST will empower people to self-test for HIV. HIVST is likely to minimize stigma; lack of counseling is likely to motivate people to use HIVST
	**Beliefs about the facilitators for performance:** Easy access of kits; availability of self-test kits; disclosure of negative results; positive results triggers action; appropriate locations for delivery of HIVST; strategies for advocacy HIVST; strategies for linkage to HIV care.
	**Beliefs about barriers to performance:** Unaffordable kit price; poverty; illiteracy; poor eye-sight; cost-benefit of HIVST; lack of HIVST policy; lack of counseling & linkage to HIV care; limitations of rapid HIV tests.
Self-efficacy	The availability of face-to-face counseling will clear doubts about an individual’s capacity to perform HIVST; correct information would increase HIVST knowledge and the capacity to perform HIVST; less confidence to use HIVST correctly when alone.

#### Ethical considerations

The study was submitted and received ethical approval from the Kilimanjaro Christian Medical College Research Ethics Committee (CREC: 884), National Institute of Medical Research, Tanzania (NIMRlHQIR.8a/Vol. IX/2454), and University of Cape Town, South Africa (HREC REF: 737). Informed written consent was sought from eligible participants before data collection. Also, respondents were informed of the study objectives, confidentiality, and that participation was voluntary, and their right to withdraw from the study at any time.

At the end of the FGDs and IDIs, participants received reimbursement of Tanzanian Shillings (TZS). 3000/= (US$ 1 = 2000 TZS) for transport fare. To uphold anonymity, all FGD and IDI participants were given code numbers, which were used during the discussions, data analysis, and reporting of results.

## Results

A total of 55 participants (FBWs = 16; MCPs = 21; Key informants =18), were involved in the study and reflects representation among participants across sex, age, education level, marital status, and previous history of HIV testing. Five FBWs who were invited to attend did not participate because of a lack of time the day scheduled for FGDs. Among FBW’s, 7 had primary education, 14 were unmarried, and 10 had ever tested for HIV with a mean age of 26 years. All MCPs who were invited to attend did participate. Among MCPs, 10 had primary education, 12 were married, and 12 had ever tested for HIV with a mean age of 31 years. Among KIs, 12 were males, 17 had secondary education, 15 were married, and 13 were employed with regular salary with a mean age of 52 years (Table 2).

**Table 2:** Characteristics of participants (*n* = 55)

	Female Bar workers n (%)	Mountain climbing porters n (%)	Key informants n (%)
**Sex**			
Male	0(0.0)	21(100.0)	12(66.7)
Female	16(100.0)	0(0.0)	6(33.3)
**Data collection methods**			
In-depth interviews (IDIs)	0(0.0)	0(0.0)	18(100.0)
Focus group discussions (FGDs)	16(100.0)	21(100.0)	0(0.0)
**Age**			
Mean age in years (range)	26 yrs. (18–34)	31 yrs. (22–48)	52 yrs. (29–77)
**Education level**			
Primary education	7(43.7)	10(47.6)	1(5.6)
Secondary education or higher	9(56.3)	11(52.4)	17(94.4)
**Marital status**			
Married	2(12.5)	12(57.1)	15(83.3)
**Employment status**			
Employed with a regular salary	16(100.0)	0(0.0)	13(72.2)
**History of HIV testing**			
Ever tested for HIV	10(62.5)	12(57.1)	N/A^a^

^a^Key informants were not asked about their previous history of HIV testing

Key informants included: one (1) HIV expert at the national level, three (3) HIV experts at the regional level, three (3) HIV experts at the district level, one (1) VCT counselors, two (2) clinicians (1 in a public facility; 1 in a private facility) four (4) influential leaders (1 = Imam; 1 = HIV NGO representative; 1 = local leader; and 1 = chairperson of community advisory board), three (3) tourist experts, and one (1) bar worker. All KIs who were invited to attend the interviews did participate. We did note some differences in accounts of attitudes, perceived norms, and personal agency related to HIVST, and HIV testing experiences between FBWs and MCPs, whereby female participants contributed more information compared to their male counterparts.

### Attitude about HIVST

According to the IBM model, attitude towards a behavior refers to experiential attitude (emotional response to the idea of engaging in a behavior), and instrumental attitude (beliefs about the outcomes of the behavior). In this study, participants expressed both experiential attitudes and instrumental attitudes towards HIVST.

#### Experiential attitudes

Male participants’ emotional responses related to the uptake of HIVST, including enjoying the freedom to test after deciding to test for HIV. This was well illustrated in the following quote from a male participant: *“I would prefer self-testing because I will have the freedom to test myself, once I have decided to test for HIV”* (FGD participant, in his 30’s).

Most female participants expressed anticipated relief to avoid needle pricks by using an oral-fluid self-test, and fear of seeing blood. This observation was well described by a female participant who said: “By *using this [oral-HIVST] … you do not need to prick yourself to get a blood sample but you collect a sample from the mouth to test for HIV. I will prefer it [oral-HIVST] because I dislike seeing blood and I also fear needle pricks*” (FGD participant, in her mid 30’s).

#### Instrumental attitudes

Female participants expressed that their ability to self- test for HIV would increase their trust and acceptability of their test result:

*“First, I will have the ability to test myself. Secondly, I will trust my HIV self-test results because I am the one who has done the test, which is different from if someone would have taken my blood or a sample from the mouth and go to test for HIV. So if I test myself whatever the test result may be I will accept it”* (FGD participant, in her 20’s).

Other positive consequences of HIVST that were anticipated by both female and male participants included privacy during testing, avoidance of long queues, reduced time spent traveling to and from health facilities, reduced time spent waiting for test results, a reduced counselor’s workload, and reduced indirect costs related to transporting to facilities. Male participants particularly valued the privacy and convenience that they anticipated was a consequence of being able to test:

*“First, I will test in the privacy of my house, and this [HIVST], will reduce the costs of transport of going to the health facility to test for HIV. Secondly, I will avoid the long queues in the health facilities, while waiting for testing services. Thirdly, it will reduce the time I would use to go to the health facility and also I will use less time to test and get my results”* (FGD participant, in his late 40’s).

Anticipated positive consequences particularly valued by female participants were reduced transport costs to visit a testing point. This was well explained by a middle-aged female participant who said: *“If I buy this kit [self-test], and test myself at home, I will reduce the cost of transport to go to test at a health facility or stand-alone clinic”* (FGD participant, in her 30’s).

Additionally, a counselor observed that HIVST might free counselors’ time previously spent on testing HIV-negative individuals and hence reduce their workload: “[…] *from our side [counselors] we will have fewer clients to attend, which will reduce the workload* ”(IDI participant 12, in her 50’s).

#### Perceived norms about HIVST

It is theorized that injunctive norms (beliefs about what significant others think one should do regarding a behavior) and descriptive norms (individual’s perception about the extent to, which significant others will perform the recommended behavior) about HIVST will influence the intention to perform HIVST. Most participants expressed perceived norms that were supportive of HIVST. None mentioned norms that were not supportive of HIVST.

#### Injunctive norms

Participants expressed a belief that people in their social environment, such as parents and peers, would approve their uptake of HIVST. The belief supportive of HIVST was well illustrated in the following quote: *“I know my mother would approve I should use HIVST to test for HIV. She always insists that I should be careful with my health because we work in a very risky environment for HIV infection”* (FGD participant, in her mid 20’s).

#### Subjective norms

Some female respondents expressed a belief that significant people such as relatives or friends would accept and take up HIVST. This positive belief was illustrated in the following quote: *“I think relatives or friends will see it [HIVST] as an additional approach that may increase testing options, which is easy to use and needs less time to know their HIV status”* (FGD participant, in her mid 20’s).

#### Personal agency toward HIVST

Personal agency refers to perceived control (environment facilitates or impedes behavior) and self-efficacy (confidence a person has in their ability to perform the behavior).

#### Control belief

Most male participants agreed that testing for HIV on their own would facilitate the uptake of HIVST. Men felt that taking control of their testing, and making positive choices derived from using the HIVST kits was a positive feature of HIVST and a first step to know one’s HIV status:

*“HIVST is very important to me because it will empower me to test for HIV whenever I decide to test. This could be like the first step to know my HIV status before I decide to go to test for HIV at a health facility or testing centers”* (FGD participant, in his 20’s).

Some key informants perceived that the introduction of HIVST would be likely to minimize the stigma associated with HIV testing, and this would facilitate uptake: “*I think HIVST will reduce stigma in the community because my results will remain my secret. I am sure no one will know my HIV status unless I decide to disclose my results*” (IDI participant 11, in his mid 40’s).

Interestingly, a male informant observed that young people may be likely motivated to use HIVST, because of lack of counseling since they have no time to wait: *“Some people may be motivated to use HIVST because of lack of counseling, particularly young people who have no time to wait*” (IDI participant 14, in his mid 50’s).

Some key informants believed that monetary incentives and the availability of social support for those who test HIV positive would facilitate acceptance of HIV testing. A male informant said: “[…] *for example provide incentives [monetary] for those who will agree to test for HIV. Also, there should be social support for those who will be HIV positives, such as diet, financial assistance, and counseling”* (IDI participant 1, in his 40’s).

Conversely, a male HIV expert mentioned monetary incentives as a potential impediment:

*“Initially, incentives may work to motivate people to use HIVST kits to know their HIV status. However, this may not be sustainable in the long run, if you consider the issue of ART adherence for example. What I believe is that clients should know that HIV testing is beneficial to their overall health status, and they would proactively seek care and treatment”* (IDI participant 15, in his late 50’s).

#### Beliefs about the facilitators for performance

Some key informants believed that the features of oral-fluid HIV self-testing that would facilitate uptake were that it would be easy to use, painless and less invasive compared with finger prick for blood-based testing. A male participant, however, perceived that the stated preference of oral-fluid test might hinder uptake of HIVST because of concerns around accuracy:

*“My concern here will be the accuracy of the results from these two samples. I am not sure if the results will be the same or different. However, from our clients' perspectives, I think most will believe the results from the blood compared with the results from a sample from the mouth (oral-fluid sample). The reason is simple- the current rapid test that we are using we collect a blood sample for testing, so clients are used to that. Another reason is their understanding that the viruses are in the blood and not in the oral-fluids! If we introduce taking a sample from the mouth (oral-fluid sample) to test for HIV, it will take time and effort to convince clients that it is possible to test HIV from a sample taken from the mouth”* (IDI participant 15, in his late 50’s).

Most female participants mentioned that features of an HIVST distribution strategy that would facilitate uptake the availability of accessible locations for distribution, interventions to advocate for HIVST and make people aware of it, and interventions to ensure and linkage from HIVST to HIV prevention, treatment, and care.

Female participants believed that easy access of HIVST kits, and disclosure of a negative HIV test result, has a positive effect in performing HIVST: *“I do not think at the hospital it will be easy. I think at the drug shop or pharmacy is much easier because I will go there at any time and buy my kit and go back home to test”* (FGD participant, in her late 20’s).

Another female participant observed: *“[…]once you test and find that you are HIV negative, then you disclose your test results to your male partner, and he would be motivated to do self-testing”* (FGD participant, in her 30’s).

Most female participants perceived the results of the self-test might facilitate linkage to care. A female discussant stated: *“If I find that I am HIV positive, I will go to the health facility to confirm my test results”* (FGD participant, in her 30’s).

Most female participants commonly cited pharmacies, or drug shops, and workplaces as appropriate locations for distribution of HIVST that might facilitate the uptake of HIVST. A young woman explained: *“I think the appropriate place should be in pharmacies or drug shops or chemists, etc.*” (FGD participant, in her mid 20’s).

Some key informants perceived that the availability of appropriate locations for, and strategies of distribution of HIVST kits would facilitate uptake of HIVST and that these included community-based distributors, integration into existing services and outreach community-based interventions, and vending machines and kiosks.

A male HIV expert elaborated: *"For HIV self-test to retain its true meaning, services must be available close to residential areas […], so delivery could be door-to-door. We have the home-based care service (HBC), and the home-based care attendants can move from house to house to supply the self-test kits"* (IDI participant 8, in his late 30’s).

Conversely, a clinician perceived that public distribution of HIVST kits may negatively impact on the uptake of HIVST:

*“What is important I think is how the HIVST kits will be distributed. If the HIVST kits will be distributed in public places where there is a possibility of other people seeing the collection of the kits, no one will be willing to come forward to collect the kits”* (IDI participant 7, in his late 50’s).

Key informants perceived that strategies for advocacy and raising awareness of HIVST, including the use of influential leaders, use of existing peer networks, and effective communication, might positively impact on the uptake of HIVST. Key informants cited the role of influential leaders in raising awareness of HIVST. A woman informant explained:

*“The community leaders should be the first group of people to be trained on HIVST in the community. The community leaders would include priests, pastors, sheiks, politicians, and the so-called: ‘influential people' –people who are respected in the community. Once they* [influential leaders] *are aware of the HIVST they will play a very important role in creating awareness in the community because they have many followers who trust them* [influential leaders]” (IDI participant 3, in her 60’s).

Finally, key informants mentioned a variety of strategies that may facilitate linkage to HIV prevention, treatment, and care following HIVST. The strategies were well described by a female HIV expert in the following quote:

*“[…]if we involve community health workers in the distribution of the HIVST test kits, then it will be easy for the same community health workers to follow-up clients who have requested for the test kits and ask them if they have tested and if they have sought care. Another alternative could be to ask clients to return the self-test kits to the pharmacy or drug shop after a certain period, for example, 3 days and recorded in a register. Another alternative could be the use of mobile applications or services such as phone calls or text messages to monitor clients and link them to HIV prevention, care, and treatment services”* (IDI participant 9, in her 50’s).

#### Beliefs about barriers to performance

A common impediment to the uptake of HIVST cited by most female participants was the cost of buying the self-test kits. A young woman explained: “[…] *most people in rural settings are poor. Therefore, if these [self-testing kits] will be sold in pharmacies, it may be difficult for most people to afford”* (FGD participant, in her 20’s).

Most key informants suggested no-cost or very low cost through government subsidies or health schemes to circumvent the cost of buying the self-test kits. A male informant explained: *“I think health insurance schemes could cover for the cost of buying the HIVST kits. For example, most porters are covered by a health insurance scheme called: Micro Health Initiative, which pays for their treatment when they fall sick”* (IDI participant 1, in his 40’s).

Another impediment to the uptake of HIVST, mentioned by some female participants was the cost-benefit of buying self-test kits or food. A woman participant said: *‘I don’t want a high-cost self-test kit, [...*.]. *So would I be willing to buy a kit instead of food? If that is the choice the question is what will I choose? Will I choose to buy the self-test kit or a loaf of bread?’* (FGD participant, in her mid 20’s).

Participants mentioned other constraints that may hinder the uptake of HIVST, including illiteracy, physical disabilities such as poor eyesight, and fear of HIV positive results. A young man explained: *‘Most people, particularly in the rural areas are illiterate; they cannot read a newspaper. How could they be able to read and follow the instructions of how to perform self-testing?’* (FGD participant, in his 20’s).

Another male participant added: ‘[…] *the problem will be to people, who have eyesight problems, then it will be difficult for them to perform HIVST, and they may need assistance’* (FGD participant, in his 20’s).

A male participant cited fear of HIV positive results:

*'Fear of HIV positive results is what makes most people not to test for HIV. The main reason for this fear is anticipated-stigma-whereby people think that if they are diagnosed HIV positive, other people will know and isolate them either at the workplace or in their community'* (IDI participant 14, in his 50’s).

Key informants perceived lack of policy on HIVST might hinder uptake. A male HIV expert had this to say: ‘[…] *lack of policy on HIVST may be a barrier to self-testing’* (IDI participant 15, in his late 50’s).

Male participants raised concerns regarding the quality of HIVST kits because of a lack of regulatory mechanisms and past experiences of buying fake drugs: *‘We have experiences of buying fake drugs from some drug shops / or pharmacies. What will prevent them not to sell fake self-testing kits?’* (FGD participant, in his 30’s).

Most HIV testing experts perceived lack of counseling, and linkage to care as another constraint to the uptake of HIVST, leading to missing or delayed initiation to treatment: ‘[…], *I anticipate lack of linkage to care, particularly for those who will test HIV positive, if there will be no follow-up mechanisms. This may lead to missing or delayed initiation of treatment’* (IDI participant 9, in her 50’s).

A female HIV testing expert expressed her concern related to the limitations of HIVST as a screening test, and a follow-up visit to a health facility for a confirmatory test in case of a reactive result:

*"My concern is the fact that HIV self-testing is a screening procedure. If my test result is reactive, then I need to go to the health facility again for a confirmatory test. My other concern is that HIVST tests like any other rapid HIV tests have a limitation of not detecting acute HIV infection during the window period"* (IDI participant 13, in her 50’s).

#### Self-efficacy towards HIVST

Most men were confident in their capacity to use the HIVST kits. Some male participants stated their preference for face-to-face counseling with a trained counselor to clear doubts about an individual’s performance with HIVST kits. A male participant explained: *“Wherever the self-test kits would be available, clients must receive counseling and a demonstration on how to use the self-test kits before they buy the kits and test for HIV. Once clients receive all the necessary information, self-testing correctly for HIV is possible”* (FGD participant, in his mid 20’s).

However, some female participants were less confident in their capacity to use the HIVST kits correctly when alone. Their lack of confidence in their capacity to use the kits may lead to potential mistakes, which would reduce their trust in the self-test results:

*“ I will not be confident to test myself as if I will go to ANGAZA (a stand-alone VCT site)…that the instrument [HIVST] has shown correctly the results…maybe I have made a certain mistake or there may be something which I have done wrong…”* (FGD participant, in her 30’s).

## Discussion

This formative study employed IBM to explore FBWs, MCPs and key informants (KIs) attitudes, perceived norms, and personal agency beliefs related to HIVST in Northern Tanzania.

### Attitudes about HIVST

Despite the lack of HIVST awareness in Tanzania, most participants expressed positive experiential, and instrumental attitudes towards HIVST. Positive attitudes towards HIVST were primarily related to enjoying the freedom to test for HIV in the privacy and at a convenient place of their choice, and relief to avoid needle pricks for using the oral-fluid sample, which is a less invasive and painless procedure. These findings are in line with studies done in other settings [44–46]. Thus, we expect that future HIVST intervention using oral-fluid samples among FBWs and MCPs is more likely to be accepted in Northern Tanzania.

In this study, health care providers expressed an anticipated positive consequence about HIVST, because used as a triage, HIVST might increase efficiency, freeing counselors’ time previously spent on HIV-negative individuals and reducing their workload. In most LMICs, such as Tanzania there is a shortage of health care providers, including counselors who are facing heavy-workloads in clinical settings, which lead to burnout syndrome, resulting in inefficiency [47]. Therefore, HIVST will enable counselors to have more time focusing on those with reactive results in need of confirmatory testing and initiation of Anti-retroviral treatment (ART) [20].

### Perceived norms about HIVST

Perceived norms about HIVST did not emerge as key issues in participants’ discourse. However, when they arose in the interviews and discussions, they were supportive of HIVST. These findings are consistent with findings from other studies [35, 48–51]. According to IBM, both injunctive and descriptive norms are important constructs of social influence in social identity and might influence the uptake of recommended behavior, such as HIVST [31, 35]. Within the HIVST context, which provides autonomy and perceived self-control of testing for HIV, participants in this study might find injunctive norms less influential in their decision-making process [31].

### Personal agency towards HIVST

In this study, most participants expressed beliefs about the facilitators for the uptake of HIVST. Some of the facilitators for the uptake of HIVST are more salient for men since evidence suggests that time-consuming activities, for example, discourage men to seek HIV testing services [52]. Also, several studies have documented men’s reluctance to go to test at an HIV testing point; because of the so-called ‘masculinity’-which is a social construct around male ego of not showing their feelings in public, and the perception that HIV testing is a woman’s domain [53–57].

Findings from this study highlight that HIVST is likely to minimize the stigma associated with HIV testing at a testing point or facility by reducing visibility associated with visiting clinical settings, lack of confidentiality of test results, and long waiting times. Besides, the perceived facilitators reported in this study (i.e., privacy, confidentiality, convenience, etc.) may also reduce the stigma related to visiting a health facility for HIV testing. This observation corroborates previous studies on stigma related to accessing HTS in clinical settings [15, 16, 18, 53, 58].

Young people perceived a lack of counseling favorable because they usually do not have time to wait long at HIV testing points. This observation is in line with findings from other settings, whereby people dislike face-to-face counseling because it is repetitive, frustrating, intrusive, and time-consuming [52]. Although mistrust of what happens to specimens collected in a clinic or other testing settings is not well documented in Tanzania, the ability to self-test for HIV was perceived to increase trust and acceptability of test results in this study. This finding is contrary to a study in the USA where some participants preferred HIV testing conducted by a professional using a blood sample [24]. Also, the different perceptions on preferences between oral-fluid (OF) specimens compared with finger stick blood (FSB) and the accuracy of results reported in this study must be addressed to correct misconceptions related to the accuracy of HIVST test results and different specimen collection methods [14–16]. Evaluation studies on the performance of the rapid test for HIVST have shown that the FSB test was more reliable than OF test, with the variability of the accuracy of results reported in different settings [59, 60]. A median sensitivity (93.6%) and specificity (99.9%) of OraQuick HIV self-test kits were reported in a global review of HIVST [61].

Since HIVST is not familiar in Tanzania, and to facilitate advocacy and awareness creation of HIVST to increase uptake of HIV testing, a peer-based approach for HIVST education and promotion could improve target population behaviors towards HIV testing. Existing evidence suggests that the use of peer-educators for the promotion of health –behaviors related interventions are highly effective in behavior change, such as HIV testing [62–67].

Different service delivery approaches for HIVST were commonly cited by most participants as potential facilitators for effective linkage to HIV prevention, care, and treatment following HIVST. For example, using of door-to-door distribution of HIVST by trained community-based distributors was viewed as an effective strategy of linkage to care, by offering a proactive follow-up, including post-test counseling, and assistance on referrals for a confirmatory test [20]. Further, the use of mobile applications, such as phone calls or short messages services (SMS) is also recommended to facilitate linkage to HIV prevention, care, and treatment. Other studies have documented the use of mobile applications as a cost-effective intervention in increasing HIV testing [68, 69]. In Tanzania, 78% of households own a mobile phone, [70], so it is feasible for future HIVST intervention to use phone calls or SMS to facilitate linkage to HIV prevention, care, and treatment among FBWs and MCPs in Northern Tanzania.

Commonly cited constraints that may hinder uptake of HIVST across the literature such as cost of buying self-test kits, limitations of self-test kits, of not detecting acute HIV infections, missing or delay treatment among HIV positive individuals that may arise following self-testing due to lack of pre and post-test counseling, and strategies for linkage to care were also reported amongst participants in this study. To circumvent constraints such as the cost of buying self-test kits, implementation of low or no cost HIVST kits, through subsidies, or insurance mechanisms to cover for the cost of buying the kits may be an alternative approach among the target population in Northern Tanzania [18, 19, 56].

### Self-efficacy towards HIVST

Our results showed mixed findings on the participant’s confidence in their capacity to use the HIVST. Interestingly, men compared to women, reported high confidence in using HIVST correctly after face-to-face counseling with a trained counselor, parallel to findings from a study conducted in Tanzania [46]. However, female participants expressed a lack of confidence in using HIVST in privacy, underscoring the importance of self-efficacy influence on an individual’s confidence in their ability to perform a new technology [56, 71]. Evidence suggests that lack of instructions-for-use, and/ or lack of demonstrations on how to perform and interpret the self-test results contribute to user errors [71]. To address the low confidence in using HIVST correctly, interventionists must consider providing additional guidance on how to accurately perform the HIVST particularly among FBWs in Northern Tanzania [56, 71].

### Limitations

The current study has limitations worth noting. The findings might be affected by selection bias because we sought to include a purposive sample of study participants, but other potentially relevant participants with different views on the study topic may not have been adequately represented in the sample. It is important to remember the limits of focus group data. While focus groups are very good at uncovering the range of experience, they are not good at uncovering how common any one experience might be. This is because not every person was asked or required to answer every question. A participant’s silence does not necessarily mean that they did not have an opinion/ or an experience.

Also, we did not assess the participant’s knowledge about HIVST, the ability to use and follow HIVST instructions or interpret HIVST results. Future studies should explore the target population’s perceptions towards HIVST after viewing different demonstration materials (e.g. videos, print materials, simulation on how to use self-test kits) to determine which methods are more effective for informing potential self-testers.

Finally, social desirability bias may be present in data collection due to the sensitive nature of the topic with participants likely to provide answers they think are socially acceptable.

## Conclusions

The findings from this study suggest that IBM can be used to develop an HIVST intervention for HIV testing among FBWs and MCPs, and may be feasible to implement in this study setting. Since HIVST is not publically used in Tanzania, it is imperative to develop awareness campaigns to increase HIVST knowledge among FBWs and MCPs. Participants’ beliefs about the potential facilitators and barriers to uptake of HIVST can guide implementation strategies. In designing HIVST interventions, it is important to take into account negative beliefs, and perceived barriers towards HIVST that might undermine the uptake of HIV testing.

## Supplementary information

**Additional file 1.** English version interview guide.

## Data Availability

The datasets generated and analyzed during this study are not publicly available but may be available from the corresponding author upon reasonable request, and with permission from the University of Cape Town, South Africa.

## Abbreviations

ART: Antiretroviral treatment
CTC: Care and Treatment Centers
FDA: Food and Drugs Administration
FBWs: Female Bar Workers
HBM: Health Belief Model
HIVST: HIV Self-Testing
HTS: HIV Testing Services
IBM: Integrated Behaviour Model
LMICs: Low and Middle-Income Countries
MCPs: Mountain Climbing Porters
NGO: Non-Governmental Organization
PLWH: People Living with HIV
SCT: Social Cognitive Theory
TPB: Theory of Planned Behavior
TRA: Theory of Reasoned Action
VCT: Voluntary Counseling and Testing
