# Supplementary material for: Feasibility of an HIV self-testing intervention: a formative qualitative study among individuals, community leaders, and HIV testing experts in northern Tanzania
Source: BMC Public Health. 2020 Apr 15;20:490. doi: 10.1186/s12889-020-08651-3 (PMC7161285; doi:10.1186/s12889-020-08651-3)
Supplement: Supplementary file 1 — Additional file 1. English version interview guide. [file 12889_2020_8651_MOESM1_ESM.docx]

| **INTERVIEW GUIDE-ENGLISH VERSION: The Project title: “The development and evaluation of a theory-informed HIV Self–testing intervention among hard to reach adults in northern Tanzania”.** | |
| --- | --- |
| 1. | **INTRODUCTION [15 min]:**  **A. Introduce yourself to the interviewee**  **1.1 Introduce in-depth interview/focus group purpose and objectives**  The main purpose of this research is to know if HIV self-testing (HIVST) can be offered to participants at high risk of HIV infection and whether this intervention would improve HIV testing. During the interview, I will ask you different questions about your thoughts on HIV testing in general and HIV self-testing in particular. Please keep in mind that *there are no right or wrong answers*, we are only interested in what you think. We will not offer HIV testing as part of this interview, and you do not have to tell us about any HIV test results you may have received previously. You may refuse to answer any questions or end your participation at any time.  **1.2 INTERVIEWER: Distribute and review the informed consent form**  Your rights as a participant in this research study are important. We would like to review the Informed Consent Form and ask you to sign it. There are 2 copies, one that we will keep and the other is yours.  ***Interviewer:***  Read the consent form verbatim  Before each new heading ask if there are any questions.  Ask to sign one copy of the form  **1.3 Collect signed consent forms and record respondent ID**  ***Interviewer:***  Collect the signed informed consent form from the interviewee.  Sign and date the consent form.  *Put a unique ID on both the consent form and the in-depth interview guide*  **Date:** _ _/_ _/_ _ _ _ |
|  | **A: PERSONAL INFORMATION [5 MIN]**  ***Participant ID:*** [ ]  *Enrolment date:*  *Interviewer:*  *Place of interview:*  *Age:*  *Gender:*  *Education level:*  *Occupation:*  *Currently working(yes/no):*  *Religion:*  *Residence:* |
|  | **B: Knowledge and attitude toward HIV testing:**  **Q1:** What is the first thing that comes to mind when you hear the phrase HIV testing?  **Q2:** Think back to when you've had HIV testing: What makes HIV testing acceptable?  **[Probe:** place of testing; motivating factors; benefits of testing; etc].  **Q3:** Think about people in your community/or your workplace who have not tested for HIV. What makes people not to test for HIV?  **[Probe:** Reasons for not testing; who are least to test in the community; ways to increase uptake of HIV testing].  **Q4:** Now I am interested in learning about your attitude towards HIV testing. What would you wish would have been different about your own testing experience?  [**Probe:** Location of testing or person doing the test; the importance of counseling; confidentiality; disclosure of test results; type of specimens(blood/or saliva), etc)]. |
|  | **C: Knowledge of HIV counseling and testing approaches.**  **Q5:** Think back to when you heard about HIV testing. What types of HIV testing and counseling approaches do you know about? In other words, what are different ways that people can get tested for HIV?  Interviewer: record all answers, and then ask only about the HIV self-testing (HIVST) approach.  **D: *HIV self-testing as a delivery service in Tanzania***  Now we would like to explore your thoughts about **HIV SELF TESTING (HIVST),** which is not currently available in Tanzania but might be possible in the future. When I talk about self-testing, I  want you to think about HIV test kits that may be available for purchase at a pharmacy, with tests performed by the client usually at a private place. |
|  | **E: Knowledge of HIVST.**  **Q6:** What is the first thing that comes to mind when you hear the phrase HIV self-testing?  [**Probe:** Source of information; availability of adequate information; different communication channels to disseminate the knowledge, etc.].  **Q7:** If we offer HIV self-testing, do you think you will be interested? Why or why not?  **F: Experiential attitude towards HIVST**  **Q8:** How do you feel about the idea of HIVST [**Probe:** what do they like/dislike about HIVST; what do they hate about HIV self-testing, etc ]  **G: Instrumental attitude towards HIVST**  **Q9:** What are the positive consequences that might result from you doing HIVST**[Probe:** advantages of doing HIVST].  **Q10:** What are the benefits that might result from doing HIVST?  [**Probe:** Privacy of testing; confidentiality of test results; reduced stigma; minimal physical contact with a counselor, fewer needle pricks, ability to get at high-risk people, increase disclosure among couples, etc.].  **Q11:** What are the negative consequences that might result from you doing HIVST**[Probe:** disadvantages of doing HIVST, fear of negative social consequences post HIV positive results; stigma & discrimination; involving other family members; etc].    **H: Normative influence on HIVST**  **Q12:** What would people who are important to you think you should do regarding HIVST?[**Probe:** spouse/live-in partner willingness to self-test; significant others willingness to self-test (e.g., father, mother, relatives, religious leaders, peers, local leaders, etc].  **Q13:** What would people we have just discussed think about HIVST?[**Probe:** spouse/live-in partner willingness to self-test; significant others willingness to self-test (e.g., father, mother, relatives, religious leaders, peers, local leaders; reasons for not using HIVST,etc.].  **I: Perceived control towards HIVST**  **Q14:** Think back to your HIV testing experiences or knowledge about HIV testing; What do you think will make it easy for you to do HIVST[**Probe:** training on HIVST; availability of HIVST kits, demonstrations on how to do HIVST, simple /clear instruction, language, etc].  **Q 15:**Think back to your HIV testing experiences or knowledge about HIV testing; What do you think will make it hard for you to do HIVST?[**Probe:** risky behaviours(e.g, alcohol/substance abuse; risky sexual behaviours (i.e, unprotected sex; transactional sex; multiple sexual partners)].  **J: Barriers to, and facilitators for HIVST**  **Q16:** Think of people who may decide to use HIV self-testing; What do you think will motivate or impede them to test for HIV?[**Probe:** lack of policy on self-testing; lack of regulatory systems for HIV self-testing; lack of knowledge on self-testing; lack of counselor assistance; lack of the self-testing kits; fear of HIV-positive results, inability to perform the testing, cost of buying self-testing kits, etc.].  **K: Self-efficacy towards HIVST**  **Q17:** How confident are you that you would self-test for HIV?[**Probe:** ways to overcome the barriers to HIVST; ways to enhance facilitators for HIVST, etc.].  **Q18:** In your opinion, how do you think an HIVST intervention should be conducted?  **[Probe:** how will this be possible? Someone gets a self-test kit, and then go to test in privacy, then what next? Would they prefer self-testing at the clinic or alternative sites? Provision of assistance from counselors? A need for post-test counseling? Confirmatory test for HIV positive results? Referral of HIV-positives to care & treatment? , the role of opinion leaders in promoting HIVST intervention, channels of communication, etc].  **Q19:** If HIVST was to be offered into the community, is there anything that makes it very difficult to promote HIVST or convince someone to use HIVST? Is there anything? |
|  | **CONCLUSION:**  **Interviewer:** Now I have asked you all my questions. Do you have additional comments about increasing uptake of HIV counseling and testing in general and HIVST in particular or advice on how best to conduct an HIVST intervention at a health facility for at high-risk adults? |
|  | **THANK YOU FOR YOUR COOPERATION?** |
